# Dietary diversity and its associations with sleep quality and chronotype in young and middle-aged adults

**DOI:** 10.3389/fnut.2025.1743065

**Published:** 2026-01-26

**Authors:** Anda Zhao, Yiting Chen, Zhen Li, Qing Fan, Jiang Wu

**Affiliations:** 1 Department of Clinical Nutrition, Huadong Hospital, Fudan University, Shanghai, China; 2 School of Public Health, Shanghai Jiao Tong University School of Medicine, Shanghai, China; 3 School of Public Health and Medical Technology, Xiamen Medical College, Xiamen, China

**Keywords:** chronotype, depression, dietary diversity, propensity score matching, sleep quality

## Abstract

**Background:**

While sleep quality and chronotype are critical to wellbeing, the role of dietary diversity remains scarcely investigated, particularly among young and middle-aged adults. This study aimed to examine the associations of dietary diversity with sleep quality and chronotype, and to explore whether depression mediates these relationships.

**Methods:**

Data were derived from the 2024–2025 China Nutrition and Sleep Survey (CNSS), including 4,128 adults aged 20–59 years. Dietary diversity indices, including total dietary diversity scores (DDS), plant-based DDS, animal-based DDS, anti-inflammatory diet diversity index (AIDDI) and protein-enriched diet diversity index (PEDDI), were calculated from food frequency questionnaires. Sleep quality, chronotype, and depression were assessed using the Pittsburgh Sleep Quality Index (PSQI), the Morningness-Eveningness Questionnaire-5 (MEQ-5), and the Patient Health Questionnaire-9 (PHQ-9), respectively. Linear and logistic regression analyses were performed, with propensity score matching (PSM) applied to reduce confounding. Mediation and interaction analyses were further conducted.

**Results:**

Higher dietary diversity indices were significantly associated with lower PSQI scores and higher MEQ-5 scores, both before and after PSM. Depression might be partially involved in the observed associations with sleep quality and chronotype. The associations between dietary diversity and sleep quality were stronger among females, older adults, non-drinkers, and those with regular exercise or depressive symptoms, whereas associations with chronotype were generally consistent across subgroups.

**Conclusions:**

Greater dietary diversity is associated with better sleep quality and earlier chronotype, with depressive symptoms potentially playing a role in explaining these associations.

## 1 Introduction

Sleep plays a crucial role in sustaining physical and mental health (1, 2). Healthy sleep is regulated by a dual process: a homeostatic mechanism dependent on prior wakefulness, and an internal circadian rhythm (3). Chronotype, representing individual variations in the preferred timing of daily activities, is strongly associated with sleep quality (4). Furthermore, both poor sleep quality and an evening chronotype are linked to a higher risk of obesity, diabetes, cardiovascular diseases, and mortality (5–12). Young and middle-aged adults, who often face substantial work and family demands, are particularly vulnerable to sleep disturbances and circadian misalignment (13).

As a modifiable component of lifestyle behaviors, diet is a major factor closely linked to sleep quality and chronotype (14, 15). Accumulating evidence has shown that specific nutrients and dietary patterns are associated with better sleep quality, longer duration and an earlier chronotype (16–19).

Dietary diversity, defined as the variety of food groups consumed over a reference period, is a widely used indicator of overall diet quality and nutrient adequacy (20). Previous studies have highlighted the beneficial effects of dietary diversity on chronic diseases as well as on healthy aging (21–23).

However, evidence regarding dietary diversity and sleep outcomes remains limited. To date, only three studies have examined the association between dietary diversity and sleep quality, primarily among elderly populations (20, 24, 25). Importantly, no study has yet explored the association between dietary diversity and chronotype; additionally, evidence among younger age groups who experience distinct lifestyle pressures and circadian challenges is still sparse.

Furthermore, depressive symptoms are closely linked to both dietary patterns and sleep-related outcomes (26, 27) and emerging evidence suggests that improvements in diet quality are associated with reduced depressive symptoms and better sleep (28). Nevertheless, whether depressive symptoms are involved in the associations between dietary diversity, sleep quality, and chronotype has not been systematically examined.

Therefore, the present study aimed to investigate the associations between dietary diversity and sleep quality and chronotype in a population-based sample of young and middle-aged adults. We further explored whether depressive symptoms might be involved in these associations and examined potential interaction effects by sociodemographic, behavioral, and psychological characteristics.

## 2 Methods

### 2.1 Study design and population

This research is part of the ongoing China Nutrition and Sleep Survey (CNSS), which incorporates both cross-sectional and cohort study designs, and is designed to explore the association between diets and sleep health. The CNSS is initiated in 2024 and conducted by Huadong Hospital. We integrated the data from the cross-sectional investigation of the 2024 wave and the 2025 wave to increase statistical power and to better capture dietary and sleep characteristics in young and middle-aged adults, the primary population of interest in this study.

The 2024 wave employed a quota sampling method to recruit 2,544 adults across China's 31 provinces between December 2024 and January 2025. To ensure representative sampling, the 31 provinces were initially stratified into seven geographic regions, and the gender and age composition of the study sample was proportionally aligned with the national demographic distributions reported in the 53rd Statistical Report on Internet Development in China (2023).

The 2025 wave adopted purposive sampling, specifically focusing on the recruitment of young and middle-aged adults (aged 20–59 years). This targeted approach aimed to gather detailed information from a key demographic segment relevant to our primary research questions on dietary behaviors and sleep. Similar to the 2024 wave, data collection for the 2025 wave was conducted between June 2025 and July 2025 across the same seven geographic regions of China, utilizing an identical questionnaire, recruiting a total of 2,537 participants.

To ensure a consistent and focused study population across both waves for the present analysis, we excluded participants who were outside the age range of 20–59 years, as well as those with missing data on either the Food Frequency Questionnaire (FFQ) or the sleep questionnaire. Survey wave was additionally adjusted for in multivariable models, as well as sensitivity analyses stratified by survey wave and wave-constrained propensity score matching (PSM) were conducted to assess the robustness of the findings (see Section 2.6 for details).

The final analytical sample comprised 4,128 participants: 1,752 from the 2024 wave and 2,376 from the 2025 wave. The participant flowchart is provided in Supplementary Figure S1. Huadong Hospital's Ethics Committee licensed our protocol (Approval No. 2024K335), and all contributors gave their consent.

### 2.2 Assessment of dietary diversity

A simplified FFQ encompassing nine major food groups: vegetables, fruits, legumes and their products, nuts, meat, eggs, fish, dairy products, tea, was used to evaluate dietary diversity. Cereals and oils were omitted from the dietary diversity assessment, as both are consumed daily by almost all Chinese individuals. The reproducibility and validity of this FFQ have been verified in prior studies (29).

Dietary diversity scores (DDS) and related indices are used to evaluate dietary diversity (21–23). The scoring approach for the DDS followed methods used in a number of previous studies conducted in Chinese populations, which applied similar intake frequency cut-offs to assign scores to each food group (30–32). Consistent with these studies, we coded intake frequencies according to the following criteria. For vegetables and fruits, intake frequency was categorized as daily, often, occasionally, or rarely. In this context, “often” referred to consumption on several days per week but not every day. Participants reporting daily or often consumption were assigned a score of 1, whereas those reporting occasionally or rarely consumption were assigned a score of 0. For the remaining food items, intake frequency was classified as daily, weekly, monthly, occasionally, or rarely; participants reporting daily or weekly consumption received a score of 1, and those with less frequent intake a score of 0. The total DDS for each participant was calculated on a scale of 0 to 9 based on this scoring system.

In addition to the total DDS, further examined animal-based and plant-based DDS, along with two specific indices: the anti-inflammatory diet diversity index (AIDDI) and the protein-enriched diet diversity index (PEDDI). The animal-based DDS included meat, fish, eggs, and dairy, with consumption frequency scored from 0 to 4. The plant-based DDS included vegetables, fruits, legumes, and nuts, with scores assigned on a 0–4 scale according to intake frequency. Following previous studies (31, 32), the AIDDI was derived with reference to the dietary inflammatory index (DII), ranging from 0 to 5. The DII is constructed based on literature up to December 2010 examining dietary effects on inflammation, and incorporates 45 dietary components related to six inflammatory biomarkers (33). In line with previous studies (31, 32), the anti-inflammatory food group in the present research, comprised vegetables, fruits, legumes and legume products, nuts, and tea. The PEDDI, ranging from 0 to 6, was calculated by summing the scores assigned to protein-rich foods: meats, fish, eggs, dairy products, nuts, and legumes (32).

### 2.3 Assessment of sleep quality and chronotype

Sleep quality was assessed using the Pittsburgh Sleep Quality Index (PSQI) (34). The scale consists of 19 self-reported items grouped into seven components, which are summed to yield a global score ranging from 0 to 21. A total score above 7 indicates poor sleep quality, with higher values reflecting worse sleep. Several validation studies conducted in Chinese populations have demonstrated that a cut-off of 7 provides superior sensitivity and specificity for identifying poor sleep quality (35, 36).

The Morning and Evening Questionnaire (MEQ) is a self-report instrument used to evaluate chronotype (37). In this study, the simplified five-item version (MEQ-5) was adopted, which has demonstrated good test-retest reliability and construct validity (37). Higher scores indicate a greater tendency toward morning type. The total score ranges from 4 to 25, classifying participants as evening type (4–11), intermediate type (12–17), or morning type (18–25).

### 2.4 Assessment of depression

The Patient Health Questionnaire-9 (PHQ-9) was applied to assess depressive symptoms (38). It includes nine items corresponding to the diagnostic criteria of the Diagnostic and Statistical Manual of Mental Disorders. Total scores (0–27) represent overall depression severity, and a cutoff of 10 or above was used to identify participants with depression.

### 2.5 Covariates

In our study, common covariates included age, sex (male or female), ethnicity (Han or others), residence (urban or rural), educational level (junior high or below, senior high, college or above), overweight/obesity (no or yes), smoking (no or yes), drinking (no or yes), and regular physical exercise (no or yes). Overweight/obesity was defined as a body mass index (BMI) ≥24 kg/m^2^. Regular physical exercise was defined as engaging in physical activity at least five times per week, with each session lasting no less than 10 min. Additionally, to account for potential period effects and variations in sampling strategies between the two data collection phases, a binary variable indicating the survey wave (2024 vs. 2025) was included as a covariate in all multivariable models.

### 2.6 Statistical analysis

Continuous variables were expressed as mean ± standard deviation (SD) for normally distributed data and as median [interquartile range (IQR)] for skewed data. Categorical variables were summarized as counts and percentages. Group differences were analyzed using the *t*-test, Mann–Whitney *U* test, or Chi-square test, as appropriate.

Linear regression models were used to estimate β and 95% confidence interval (CI) for the association between various dietary diversity indices and PSQI or PHQ-9 score. Binary and multinomial logistic regression analyses were performed to estimate the odds ratios (OR) and 95% CI for poor sleep quality, depression, and chronotype, respectively.

PSM was performed to minimize potential confounding inherent in observational data. Propensity scores were estimated through logistic regression including age, sex, ethnicity, residence, education level, overweight/obesity status, smoking, drinking, and physical exercise as covariates. Crucially, to control for wave-specific effects, matching was performed with the strict requirement that participants were matched only within the same survey wave. This matching process was implemented using the MatchIt package in *R*, specifying the method = “optimal” argument, which seeks to minimize the average absolute distance across all matched pairs to achieve the best overall balance between the poor and good sleep quality groups. To evaluate the effectiveness of the matching, we assessed the balance of covariates between the groups both before and after the procedure. The standardized mean difference (SMD) was used as the primary metric for diagnosis, with an absolute SMD value of less than 0.1 considered indicative of negligible imbalance. Additionally, visual inspection of the distribution of propensity scores plots was conducted to ensure comparability between the matched groups. Subsequently, conditional binary and multinomial logistic regression models were applied to account for the matched design.

The potential mediation effects of depression on the associations between various dietary diversity indices and PSQI or MEQ-5 scores were estimated by mediation models. Mediation analyses used the non-parametric bootstrap method with 1,000 simulations to calculate the direct effect, indirect effect, and mediation proportion.

Sensitivity analyses were conducted to verify the robustness of our results: (1) we performed regression analyses separately for the 2024 and 2025 waves to examine whether the associations remained consistent across different sampling periods; (2) we adjusted the threshold for defining poor sleep quality, with the PSQI cutoff for poor sleep quality being lowered from definition of >7 to >5.

Stratified analyses with interaction tests were further conducted, according to age, sex, overweight/obesity status, smoking, drinking, physical exercise and depression. R 4.3.2 software was applied for all statistical analyses. A two-sided *P* value of <0.05 was considered statistically significant.

## 3 Results

### 3.1 Participant characteristics

Table 1 presents the sociodemographic and lifestyle characteristics of the participants according to sleep quality before and after PSM. Before matching, a total of 4,128 participants were included, with 1,773 (42.95%) classified as having good sleep quality and 2,355 (57.05%) as having poor sleep quality. Participants with poor sleep quality were slightly younger (30.39 ± 10.73 years) than those with good sleep quality (31.71 ± 11.02 years, *P* < 0.001). Females (55.16%) and rural residents (42.25%) were more prevalent among individuals with poor sleep quality compared with those reporting good sleep (46.25 and 30.91%, respectively; both *P* < 0.001). There were no significant differences between groups in educational level, overweight/obesity status, or physical exercise. However, participants with poor sleep quality were more likely to be smokers (22.21 vs. 18.67%, *P* = 0.006) and drinkers (60.08 vs. 49.46%, *P* < 0.001). Additionally, significant differences in sociodemographic and lifestyle characteristics were observed between the two survey waves (Supplementary Table S1).

**Table 1:** Characteristics of the total sample and propensity score-matched sample.

**Characteristics**	**Before propensity 1:1 matching**					**After propensity 1:1 matching**				
	**Total (*****n*** = **4,128)**	**Good SQ (*****n*** = **1,773)**	**Poor SQ (*****n*** = **2,355)**	**SMD**	***P-*** **value**	**Total (*****n*** = **3,546)**	**Good SQ (*****n*** = **1,773)**	**Poor SQ (*****n*** = **1,773)**	**SMD**	***P-*** **value**
Age; mean ± SD	30.96 ± 10.87	31.71 ± 11.02	30.39 ± 10.73	0.122	<0.001	31.57 ± 11.10	31.71 ± 11.02	31.43 ± 11.19	0.025	0.452
Sex; n (%)				0.179	<0.001				0.056	0.099
Male	2,009 (48.67)	953 (53.75)	1,056 (44.84)			1,856 (52.34)	953 (53.75)	903 (50.93)		
Female	2,119 (51.33)	820 (46.25)	1,299 (55.16)			1,690 (47.66)	820 (46.25)	870 (49.07)		
Ethnicity; *n* (%)				0.129	<0.001				0.048	0.178
Han	3,827 (92.71)	1,677 (94.59)	2,150 (91.30)			3,334 (94.02)	1,677 (94.59)	1,657 (93.46)		
Others	301 (7.29)	96 (5.41)	205 (8.70)			212 (5.98)	96 (5.41)	116 (6.54)		
Residence; *n* (%)				0.237	<0.001				0.079	0.020
Urban	2,585 (62.62)	1,225 (69.09)	1,360 (57.75)			2,384 (67.23)	1,225 (69.09)	1,159 (65.37)		
Rural	1,543 (37.38)	548 (30.91)	995 (42.25)			1,162 (32.77)	548 (30.91)	614 (34.63)		
Educational level; *n* (%)				0.025	0.734				0.021	0.824
Junior high or below	140 (3.39)	57 (3.21)	83 (3.52)			119 (3.36)	57 (3.21)	62 (3.50)		
Senior high	415 (10.05)	184 (10.38)	231 (9.81)			375 (10.58)	184 (10.38)	191 (10.77)		
College or above	3,573 (86.56)	1,532 (86.41)	2,041 (86.67)			3,052 (86.07)	1,532 (86.41)	1,520 (85.73)		
Overweight/obesity; *n* (%)				0.039	0.232				0.015	0.693
No	3,125 (75.70)	1,359 (76.65)	1,766 (74.99)			2,707 (76.34)	1,359 (76.65)	1,348 (76.03)		
Yes	1,003 (24.30)	414 (23.35)	589 (25.01)			839 (23.66)	414 (23.35)	425 (23.97)		
Smoking; *n* (%)				0.088	0.006				0.064	0.064
No	3,274 (79.31)	1,442 (81.33)	1,832 (77.79)			2,839 (80.06)	1,442 (81.33)	1,397 (78.79)		
Yes	854 (20.69)	331 (18.67)	523 (22.21)			707 (19.94)	331 (18.67)	376 (21.21)		
Drinking; *n* (%)				0.215	<0.001				0.077	0.024
No	1,836 (44.48)	896 (50.54)	940 (39.92)			1,724 (48.62)	896 (50.54)	828 (46.70)		
Yes	2,292 (55.52)	877 (49.46)	1,415 (60.08)			1,822 (51.38)	877 (49.46)	945 (53.30)		
Regular physical exercise; *n* (%)				0.026	0.430				0.062	0.070
No	2,962 (71.75)	1,284 (72.42)	1,678 (71.25)			2,518 (71.01)	1,284 (72.42)	1,234 (69.60)		
Yes	1,166 (28.25)	489 (27.58)	677 (28.75)			1,028 (28.99)	489 (27.58)	539 (30.40)		

SQ, sleep quality; SMD, standardized mean difference; SD, standard deviation.

After 1:1 PSM, 3,546 participants (1,773 with good and 1,773 with poor sleep quality) were retained. All included covariates were well-balanced between the two groups, as indicated by absolute SMD <0.1 (Table 1) and by the comparable distributions of propensity scores (Supplementary Figure S2). Group differences in age, sex, ethnicity, and most lifestyle factors were no longer statistically significant (*P* > 0.05).

### 3.2 Association between dietary diversity and PSQI scores/poor sleep quality

As shown in Table 2, higher dietary diversity was significantly associated with better sleep quality both before and after PSM. Before matching, total DDS was inversely related to PSQI scores (adjusted β = −0.53, 95% CI: −0.62 to −0.44) and poor sleep quality (adjusted OR = 0.85, 95% CI: 0.82–0.88). After matching, these associations remained robust (adjusted β = −0.54, 95% CI: −0.64 to −0.44; adjusted OR = 0.86, 95% CI: 0.82–0.91). Similar inverse relationships were observed for animal-based DDS, plant-based DDS, AIDDI, and PEDDI. Participants with higher scores in these indices consistently showed lower PSQI scores and reduced odds of poor sleep (all *P* < 0.001).

**Table 2:** Association between dietary diversity and PSQI scores/poor SQ.

**Variables**	**Before propensity 1:1 matching**				**After propensity 1:1 matching**			
	**PSQI scores**		**Poor SQ**		**PSQI scores**		**Poor SQ**	
	β **(95% CI)**	**a**β **(95% CI)**^#^	**OR (95% CI)**	**aOR (95% CI)** ^#^	β **(95% CI)**	**a**β **(95% CI)**^#^	**OR (95% CI)**	**aOR (95% CI)** ^#^
Total DDS, per 1-score increase	−0.51 (−0.60, −0.42)^***^	−0.53 (−0.62, −0.44)^***^	0.85 (0.82, 0.88)^***^	0.85 (0.82, 0.88)^***^	−0.47 (−0.57, −0.38)^***^	−0.54 (−0.64, −0.44)^***^	0.86 (0.83, 0.89)^***^	0.86 (0.82, 0.91)^***^
Animal-based DDS, per 1-score increase	−0.87 (−1.03, −0.72)^***^	−0.84 (−0.99, −0.68)^***^	0.81 (0.76, 0.86)^***^	0.81 (0.75, 0.86)^***^	−0.79 (−0.96, −0.62)^***^	−0.83 (−1.00, −0.66)^***^	0.82 (0.77, 0.88)^***^	0.80 (0.73, 0.88)^***^
Plant-based DDS, per 1-score increase	−0.62 (−0.77, −0.47)^***^	−0.69 (−0.84, −0.54)^***^	0.77 (0.73, 0.82)^***^	0.78 (0.73, 0.83)^***^	−0.61 (−0.77, −0.45)^***^	−0.72 (−0.89, −0.56)^***^	0.78 (0.73, 0.84)^***^	0.81 (0.74, 0.88)^***^
AIDDI, per 1-score increase	−0.48 (−0.61, −0.36)^***^	−0.60 (−0.72, −0.48)^***^	0.80 (0.77, 0.84)^***^	0.81 (0.77, 0.85)^***^	−0.45 (−0.59, −0.32)^***^	−0.61 (−0.75, −0.47)^***^	0.82 (0.78, 0.87)^***^	0.84 (0.78, 0.90)^***^
PEDDI, per 1-score increase	−0.55 (−0.66, −0.44)^***^	−0.51 (−0.62, −0.40)^***^	0.87 (0.83, 0.90)^***^	0.86 (0.82, 0.90)^***^	−0.49 (−0.61, −0.38)^***^	−0.50 (−0.62, −0.39)^***^	0.87 (0.83, 0.91)^***^	0.86 (0.81, 0.92)^***^

PSQI, Pittsburgh Sleep Quality Index; SQ, sleep quality; DDS, dietary diversity score; AIDDI, Anti-inflammatory dietary diversity index; PEDDI, protein-enriched dietary diversity index; ^#^Adjusted for age, sex, ethnicity, residence, educational level, overweight/obesity, smoking, drinking, regular physical exercise and wave.

^***^P < 0.001.

### 3.3 Association between dietary diversity and MEQ-5 scores/chronotype

Table 3 shows the associations between dietary diversity indices and chronotype, assessed by MEQ-5 scores. In general, higher dietary diversity was significantly associated with a tendency toward morningness and a reduced likelihood of evening chronotype, both before and after PSM. Before matching, total DDS was positively associated with MEQ-5 scores (adjusted β = 0.29, 95% CI: 0.23–0.34), indicating an earlier chronotype. After matching, the associations remained stable. Each one-score increase in total DDS corresponded to higher MEQ-5 scores (adjusted β = 0.28, 95% CI: 0.22–0.34) and lower odds of evening type (adjusted OR = 0.75, 95% CI: 0.70–0.81). Consistent results were also found for other dietary indices. Higher animal-based DDS, plant-based DDS, AIDDI, and PEDDI were all significantly linked to higher MEQ-5 scores and lower odds of evening chronotype.

**Table 3:** Association between dietary diversity and MEQ-5 scores/chronotype.

**Variables**	**MEQ-5 scores**		**Intermediate type vs. Morning type**		**Evening type vs. Morning type**	
	β **(95% CI)**	**a**β **(95% CI)**^#^	**OR (95% CI)**	**aOR (95% CI)** ^#^	**OR (95% CI)**	**aOR (95% CI)** ^#^
**Before propensity 1:1 matching**						
Total DDS, per 1-score increase	0.38 (0.32, 0.44)^***^	0.29 (0.23, 0.34)^***^	0.78 (0.75, 0.82)^***^	0.81 (0.77, 0.85)^***^	0.70 (0.66, 0.74)^***^	0.73 (0.69, 0.78)^***^
Animal-based DDS, per 1-score increase	0.37 (0.27, 0.47)^***^	0.32 (0.22, 0.42)^***^	0.72 (0.66, 0.78)^***^	0.73 (0.67, 0.80)^***^	0.69 (0.62, 0.76)^***^	0.69 (0.62, 0.77)^***^
Plant-based DDS, per 1-score increase	0.71 (0.62, 0.81)^***^	0.50 (0.41, 0.59)^***^	0.70 (0.65, 0.76)^***^	0.75 (0.69, 0.82)^***^	0.53 (0.48, 0.58)^***^	0.61 (0.55, 0.67)^***^
AIDDI, per 1-score increase	0.65 (0.57, 0.73)^***^	0.43 (0.35, 0.51)^***^	0.72 (0.68, 0.77)^***^	0.78 (0.73, 0.83)^***^	0.56 (0.52, 0.61)^***^	0.65 (0.59, 0.71)^***^
PEDDI, per 1-score increase	0.25 (0.18, 0.32)^***^	0.24 (0.17, 0.31)^***^	0.81 (0.76, 0.85)^***^	0.81 (0.76, 0.85)^***^	0.78 (0.73, 0.84)^***^	0.77 (0.71, 0.83)^***^
**After propensity 1:1 matching**						
Total DDS, per 1-score increase	0.35 (0.29, 0.42)^***^	0.28 (0.22, 0.34)^***^	0.80 (0.76, 0.84)^***^	0.82 (0.78, 0.86)^***^	0.73 (0.68, 0.77)^***^	0.75 (0.70, 0.81)^***^
Animal-based DDS, per 1-score increase	0.33 (0.22, 0.44)^***^	0.31 (0.20, 0.41)^***^	0.73 (0.67, 0.79)^***^	0.74 (0.68, 0.81)^***^	0.73 (0.65, 0.81)^***^	0.73 (0.64, 0.82)^***^
Plant-based DDS, per 1-score increase	0.68 (0.58, 0.78)^***^	0.49 (0.39, 0.60)^***^	0.71 (0.66, 0.78)^***^	0.76 (0.70, 0.83)^***^	0.55 (0.50, 0.61)^***^	0.62 (0.55, 0.69)^***^
AIDDI, per 1-score increase	0.62 (0.54, 0.71)^***^	0.43 (0.34, 0.51)^***^	0.74 (0.69, 0.79)^***^	0.79 (0.74, 0.85)^***^	0.58 (0.53, 0.63)^***^	0.66 (0.60, 0.72)^***^
PEDDI, per 1-score increase	0.23 (0.15, 0.31)^***^	0.24 (0.16, 0.31)^***^	0.82 (0.77, 0.87)^***^	0.82 (0.77, 0.87)^***^	0.81 (0.75, 0.87)^***^	0.80 (0.73, 0.86)^***^

PSQI, Pittsburgh sleep quality index; MEQ-5, Morning and Evening Questionnaire-5; DDS, dietary diversity score; AIDDI, Anti-inflammatory dietary diversity index; PEDDI, protein-enriched dietary diversity index.

^#^Adjusted for age, sex, ethnicity, residence, educational level, overweight/obesity, smoking, drinking, regular physical exercise and wave.

^***^P < 0.001.

### 3.4 Association between dietary diversity and PHQ-9 scores/depression

As shown in Supplementary Table S2, higher dietary diversity was significantly associated with lower depression scores and reduced odds of depression, both before and after PSM. Before matching, total DDS showed a strong inverse relationship with PHQ-9 scores (adjusted β = −0.62, 95% CI:−0.71 to−0.54) and with depression (adjusted OR = 0.75, 95% CI: 0.71–0.79). After matching, the associations remained robust (adjusted β = −0.63, 95% CI:−0.73 to−0.54; adjusted OR = 0.77, 95% CI: 0.70–0.84). Similar inverse associations were observed for all sub-indices, including animal-based DDS, plant-based DDS, AIDDI, and PEDDI. Participants with higher scores in these indices had lower PHQ-9 scores and lower odds of depression (all *P* < 0.001).

### 3.5 Stratified and interaction analyses of the associations between dietary diversity and PSQI scores

**Figure 1:**
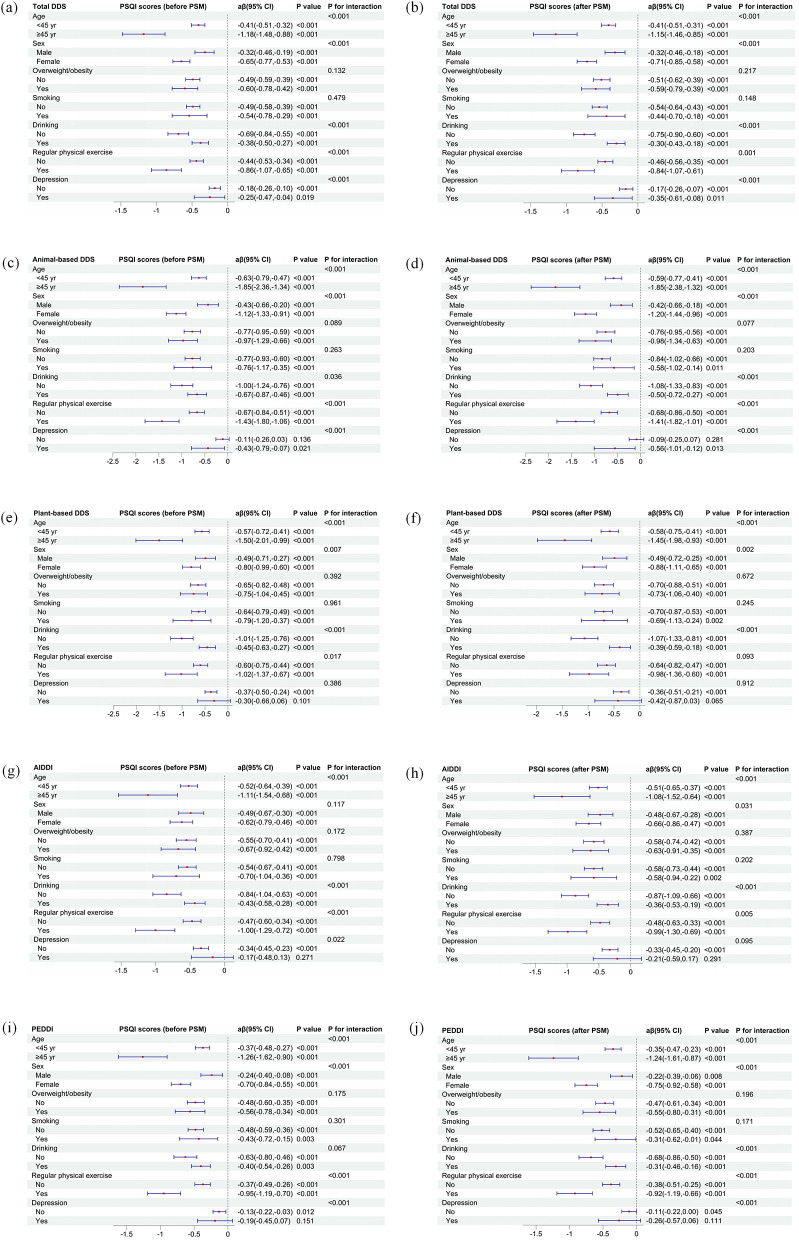
Associations of dietary diversity with PSQI scores across subgroups before and after PSM. **(a, b)** Total DDS; **(c, d)** animal-based DDS; **(e, f)** plant-based DDS; **(g, h)** AIDDI; **(i, j)** PEDDI. Adjusted for age, sex, ethnicity, residence, educational level, overweight/obesity, smoking, drinking, regular physical exercise and wave.

Figure 1 illustrates the stratified associations between different dietary diversity indices and PSQI scores before and after PSM, across subgroups of age, sex, overweight/obesity, smoking, drinking, physical exercise, and depression status. Across all indices, including total DDS (Figures 1a, b), animal-based DDS (Figures 1c, d), plant-based DDS (Figures 1e, f), AIDDI (Figures 1g, h), and PEDDI (Figures 1i, j), higher dietary diversity was consistently associated with lower PSQI scores in nearly all subgroups.

The negative associations between dietary diversity and poor sleep appeared stronger among participants aged ≥45 years, females, non-drinkers, those engaging in regular physical exercise, and those with depression. Notably, significant interactions were observed for age, sex, drinking, regular physical exercise and depression status across most indices (*P* for interaction <0.05).

### 3.6 Stratified and interaction analyses of the associations between dietary diversity and MEQ-5 scores

**Figure 2:**
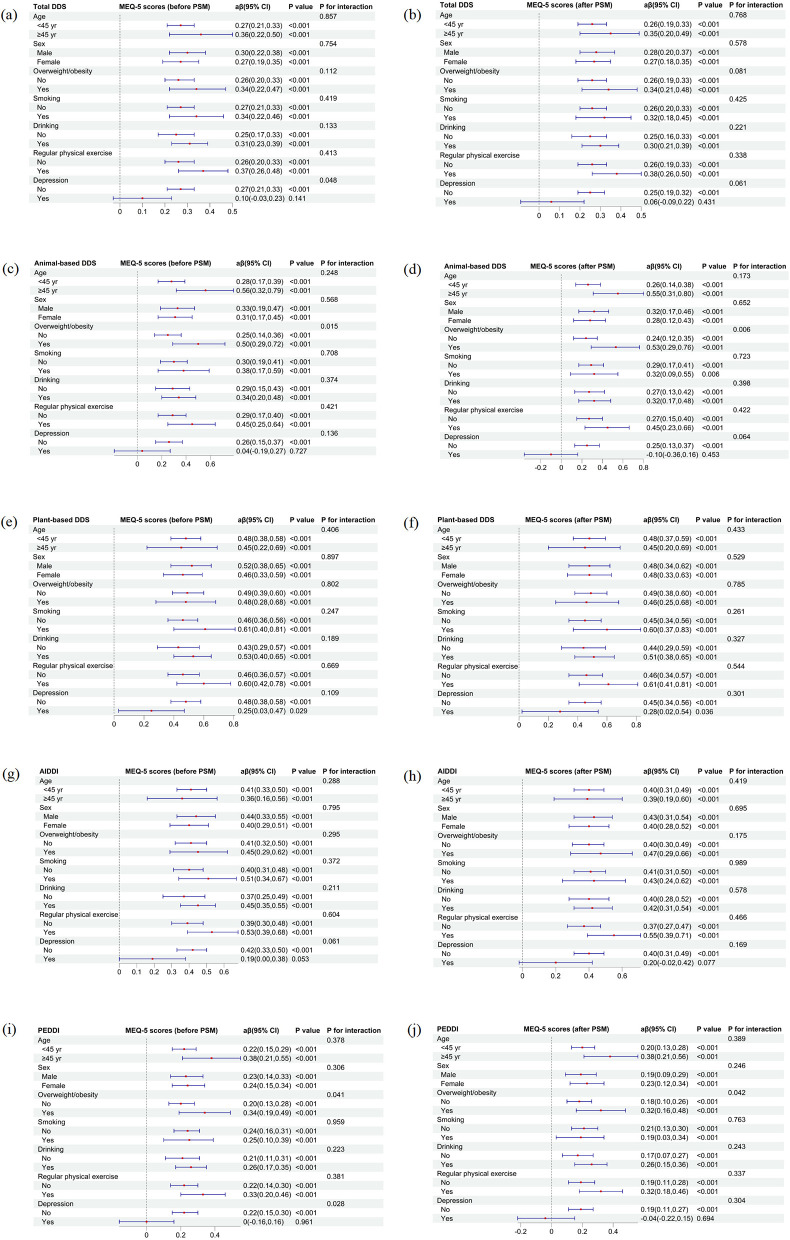
Associations of dietary diversity with MEQ-5 scores across subgroups before and after PSM. **(a, b)** Total DDS; **(c, d)** animal-based DDS; **(e, f)** plant-based DDS; **(g, h)** AIDDI; **(i, j)** PEDDI. Adjusted for age, sex, ethnicity, residence, educational level, overweight/obesity, smoking, drinking, regular physical exercise and wave.

Figure 2 presents the stratified and interaction analyses of the associations between various dietary diversity indices and MEQ-5 scores before and after PSM. Across all indices, including total DDS (Figures 2a, b), animal-based DDS (Figures 2c, d), plant-based DDS (Figures 2e, f), AIDDI (Figures 2g, h), and PEDDI (Figures 2i, j), higher dietary diversity was consistently associated with higher MEQ-5 scores, indicating a stronger tendency toward morning chronotype.

Overall, the positive associations between dietary diversity and MEQ-5 scores were stable across subgroups of age, sex, smoking, drinking, and physical activity, with no significant interactions in most cases (*P* for interaction >0.05). However, significant interactions were observed between overweight/obesity status and both animal-based DDS and PEDDI (*P* for interaction <0.05). The associations of these two indices with MEQ-5 scores were stronger among overweight/obese participants. In addition, interactions with depression status were significant in total DDS and PEDDI before PSM but disappeared after matching.

### 3.7 Mediation analyses of the associations between dietary diversity and PSQI scores

**Figure 3:**
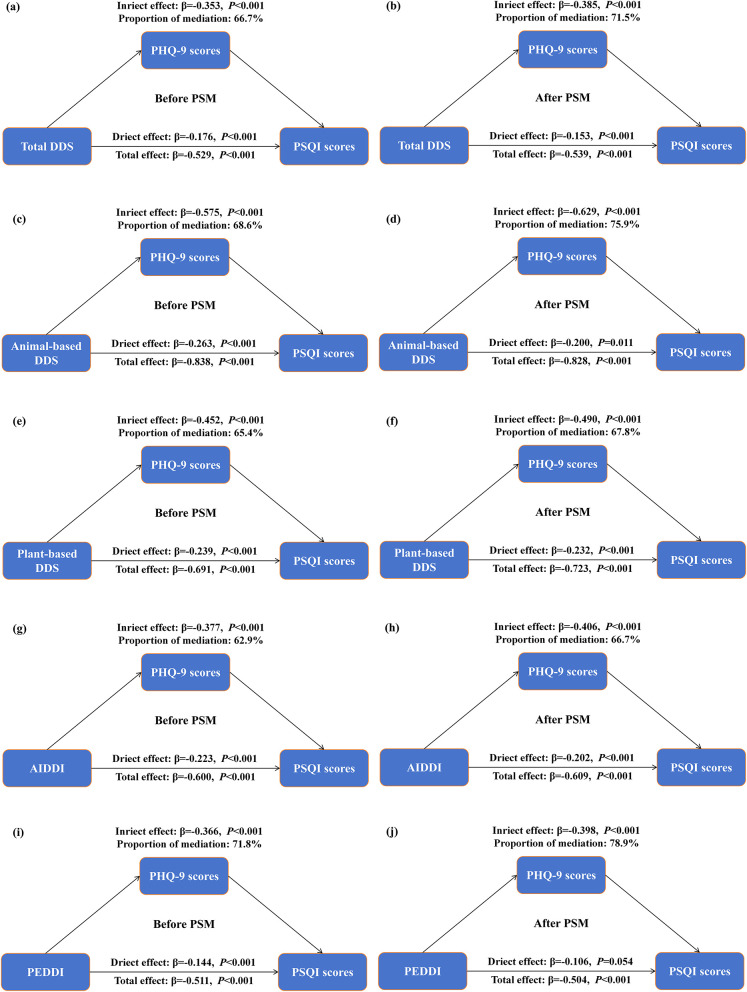
Mediation analysis of dietary diversity indices on the relationship between PHQ-9 and PSQI scores before and after PSM. **(a, b)** Total DDS; **(c, d)** animal-based DDS; **(e, f)** plant-based DDS; **(g, h)** AIDDI; **(i, j)** PEDDI. Adjusted for age, sex, ethnicity, residence, educational level, overweight/obesity, smoking, drinking, regular physical exercise and wave.

Figure 3 presents the results of the mediation analyses examining the role of PHQ-9 scores in the associations between dietary diversity and PSQI scores. Across all dietary diversity indices, higher diversity was associated with lower PSQI scores before and after PSM. The estimated indirect associations involving PHQ-9 were statistically significant. These findings suggest that depressive symptoms may be involved in the observed associations between dietary diversity and sleep quality; however, causal inference cannot be established given the cross-sectional design.

### 3.8 Mediation analyses of the associations between dietary diversity and MEQ-5 scores

**Figure 4:**
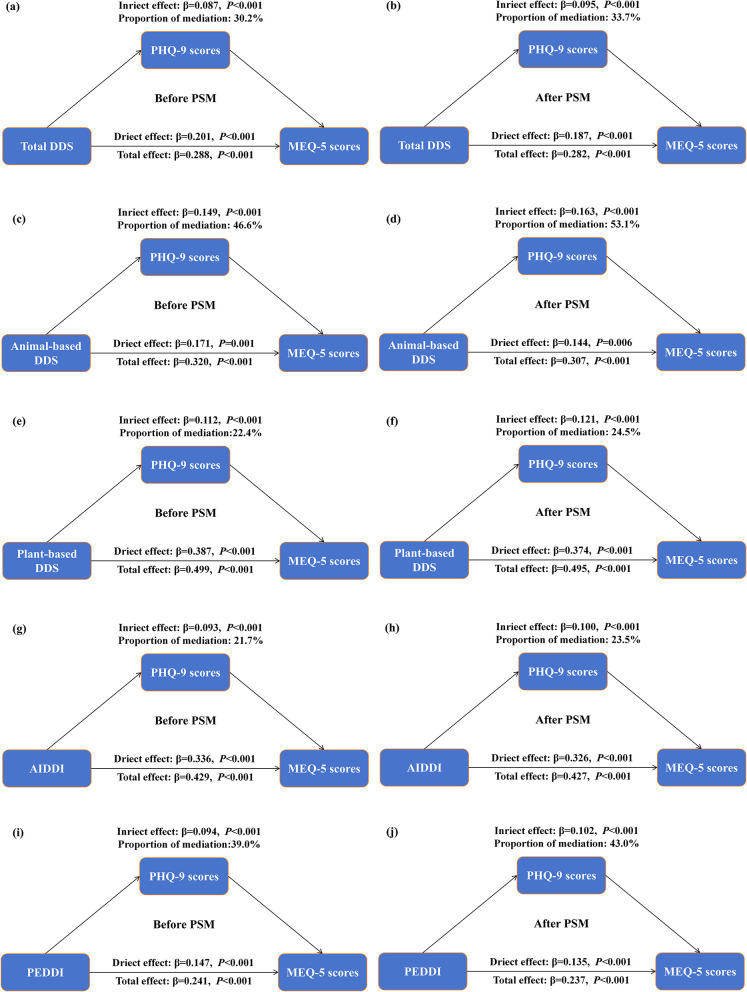
Mediation analysis of dietary diversity indices on the relationship between PHQ-9 and MEQ-5 scores before and after PSM. **(a, b)** Total DDS; **(c, d)** animal-based DDS; **(e, f)** plant-based DDS; **(g, h)** AIDDI; **(i, j)** PEDDI. Adjusted for age, sex, ethnicity, residence, educational level, overweight/obesity, smoking, drinking, regular physical exercise and wave.

Figure 4 shows that higher diversity were associated with higher MEQ-5 scores, reflecting a tendency toward a morningness chronotype. The estimated indirect associations involving PHQ-9 were significant across all indices. These findings suggest that depressive symptoms may be involved in the observed associations between dietary diversity and chronotype. The overall pattern and magnitude of these indirect associations remained consistent after PSM.

### 3.9 Sensitivity analyses

Sensitivity analyses consistently supported the robustness of the main findings (Supplementary Tables S3–S6). It showed that higher dietary diversity indices were steadily associated with lower PSQI scores, reduced odds of poor sleep quality, higher MEQ-5 scores and lower odds of depressive symptoms in both survey waves. The magnitude and direction of associations were largely comparable across waves.

When redefining poor sleep quality using the internationally applied PSQI >5 threshold, the inverse associations between dietary diversity and poor sleep quality remained significant and directionally unchanged, both before and after pooling waves and PSM. These consistent results confirm the robustness of the observed relationships.

## 4 Discussion

In this large population-based study of young and middle-aged adults in China, greater dietary diversity was associated with better sleep quality and a tendency toward chronotype. These associations were generally consistent across multiple dietary diversity indices. Results from the mediation analyses suggested that depressive symptoms might be involved in the observed associations; however, given the cross-sectional design, these findings are exploratory and require confirmation in longitudinal research.

Our findings regarding sleep quality are consistent with previous studies conducted among older populations in China and Japan, which reported associations between higher dietary diversity and better sleep outcomes (20, 24, 25). Extending this evidence, our study demonstrates that similar associations are also present among young and middle-aged adults, a population that has been insufficiently investigated despite facing substantial sleep problems. Moreover, by applying multiple complementary dietary diversity indices, we provide a more comprehensive assessment of dietary diversity and showed that the observed associations were broadly robust across different scoring approaches.

We further revealed that the associations between dietary diversity and sleep quality were more pronounced among females, middle-aged adults, non-drinkers, physically active individuals and those with depressive symptoms. These findings suggest potential effect modification by demographic and behavioral factors and underscore the importance of considering population heterogeneity when examining diet-sleep relationships.

While previous studies have separately reported associations between dietary diversity and depression (39), as well as between depression and sleep outcomes (40), the possible involvement of depressive symptoms in the association between dietary diversity and sleep quality has not been explicitly explored. Our findings provide preliminary evidence suggesting that depression might be relevant to the observed associations between dietary diversity and sleep quality; however, causal inference cannot be established due to the cross-sectional design.

Notably, our study is the first to explore the association between dietary diversity and chronotype. It showed that higher dietary diversity was associated with greater morningness preference, particularly among individuals with overweight or obesity for animal-based DDS and PEDDI. This finding is supported by recent chrononutrition evidence showing that those with morning-chronotype are more likely to adhere to nutritionally richer dietary patterns, whereas those with evening chronotype tend to have less favorable micronutrient profiles (41). Although direct evidence is lacking, several mechanisms may plausibly explain this association. First, diverse diets are more likely to provide a balanced intake of nutrients and bioactive compounds that are essential for circadian rhythm regulation (42, 43). Second, individuals with higher dietary diversity may be more likely to engage in regular eating patterns and healthier lifestyle behaviors, which are closely interconnected with chronotype and sleep quality and have been consistently associated with earlier sleep timing (44, 45). Third, greater dietary diversity has been linked with a healthier gut microbiota composition, which has been increasingly recognized as an important modulator of circadian rhythms through the gut-brain axis (46, 47). Moreover, in individuals with overweight or obesity, the presence of increased oxidative stress and inflammation may impair circadian regulation, whereas diverse protein sources have been associated with reduced inflammation and metabolic risk, potentially supporting better circadian alignment (48–50).

The present study possesses several notable strengths. First, it is the first large-scale investigation to comprehensively examine the associations between dietary diversity, sleep quality, and chronotype among young and middle-aged adults, a population that has been neglected in previous research. Second, we assessed dietary diversity using a range of validated indices (total, plant-based, and animal-based DDS, as well as AIDDI and PEDDI), providing a multidimensional assessment. Third, the use of PSM strengthened the robustness of the findings by minimizing potential confounding from sociodemographic and lifestyle variables. Fourth, the use of mediation analysis allowed us to explore the potential involvement of depressive symptoms in the associations between dietary diversity, sleep quality, and chronotype. Finally, interaction analyses provided insight into potential effect modification across demographic, behavioral, and obesity-related subgroups.

However, several limitations should also be acknowledged. First, the cross-sectional design limits causal inference, making it impossible to determine the temporal direction of the observed associations. Although mediation analyses were conducted, these analyses cannot establish causal mediation due to the lack of temporal ordering; therefore, the observed indirect associations should be interpreted as exploratory and hypothesis-generating rather than causal. Future longitudinal and interventional studies are required to clarify temporal relationships and causal pathways. Second, potential selection bias cannot be fully excluded. The integration of data from two survey waves with different sampling strategies and target populations may have introduced systematic differences in participant characteristics. Although we performed separate wave analyses and wave-constrained PSM and observed generally consistent results, residual selection bias related to participant recruitment and survey participation may still influence the observed associations. Third, dietary diversity and sleep parameters were assessed using self-reported questionnaires, which are subject to recall and reporting bias. The absence of objective measurements, such as actigraphy or polysomnography for sleep assessment and weighed dietary records or nutritional biomarkers for dietary intake, may have resulted in measurement error, potentially affecting the associations. Fourth, although we adjusted for a wide range of covariates, residual confounding from unmeasured factors cannot be entirely ruled out. Fifth, the sample composition was limited to Chinese adults, which may restrict the generalizability of findings to other cultural or dietary contexts. Lastly, although the PHQ-9 provided a reliable screening for depressive symptoms, other psychological factors such as anxiety and stress were not included and warrant further investigation.

## 4 Conclusions

In conclusion, this large, population-based study among young and middle-aged Chinese adults provides novel evidence that greater dietary diversity is significantly associated with better sleep quality and an earlier chronotype. Exploratory mediation analyses further suggested that depressive symptoms may involved in these observed associations, highlighting the potential relevance of psychological wellbeing in the links between dietary patterns, sleep, and circadian preferences. Collectively, these findings support the notion that maintaining a varied diet may be a feasible and sustainable lifestyle practice related to sleep health, psychological status, and circadian alignment in working-age adults. Future longitudinal and interventional studies incorporating objective sleep and dietary assessments are warranted to establish causality and elucidate the biological and behavioral pathways underlying these relationships.

## Data Availability

The raw data supporting the conclusions of this article will be made available by the authors, without undue reservation.
