# Supplementary material for: Dietary diversity and its associations with sleep quality and chronotype in young and middle-aged adults
Source: Front Nutr. 2026 Jan 26;12:1743065. doi: 10.3389/fnut.2025.1743065 (PMC12883360; doi:10.3389/fnut.2025.1743065)
Supplement: Supplementary file 1 [file Table_1.docx]

**Supplementary Materials**

**
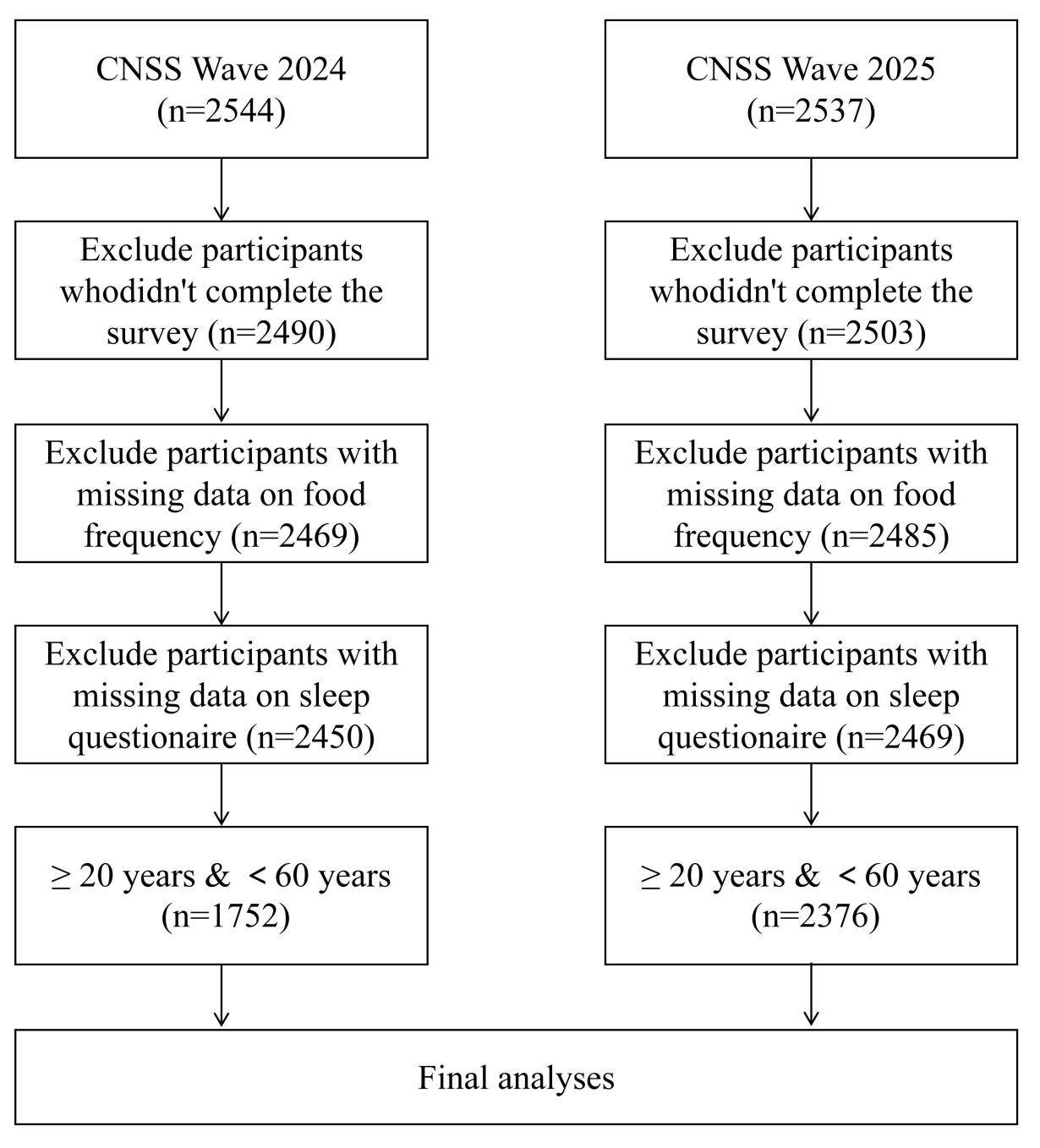
**

**Figure S1** Flowchart of the study participants

**
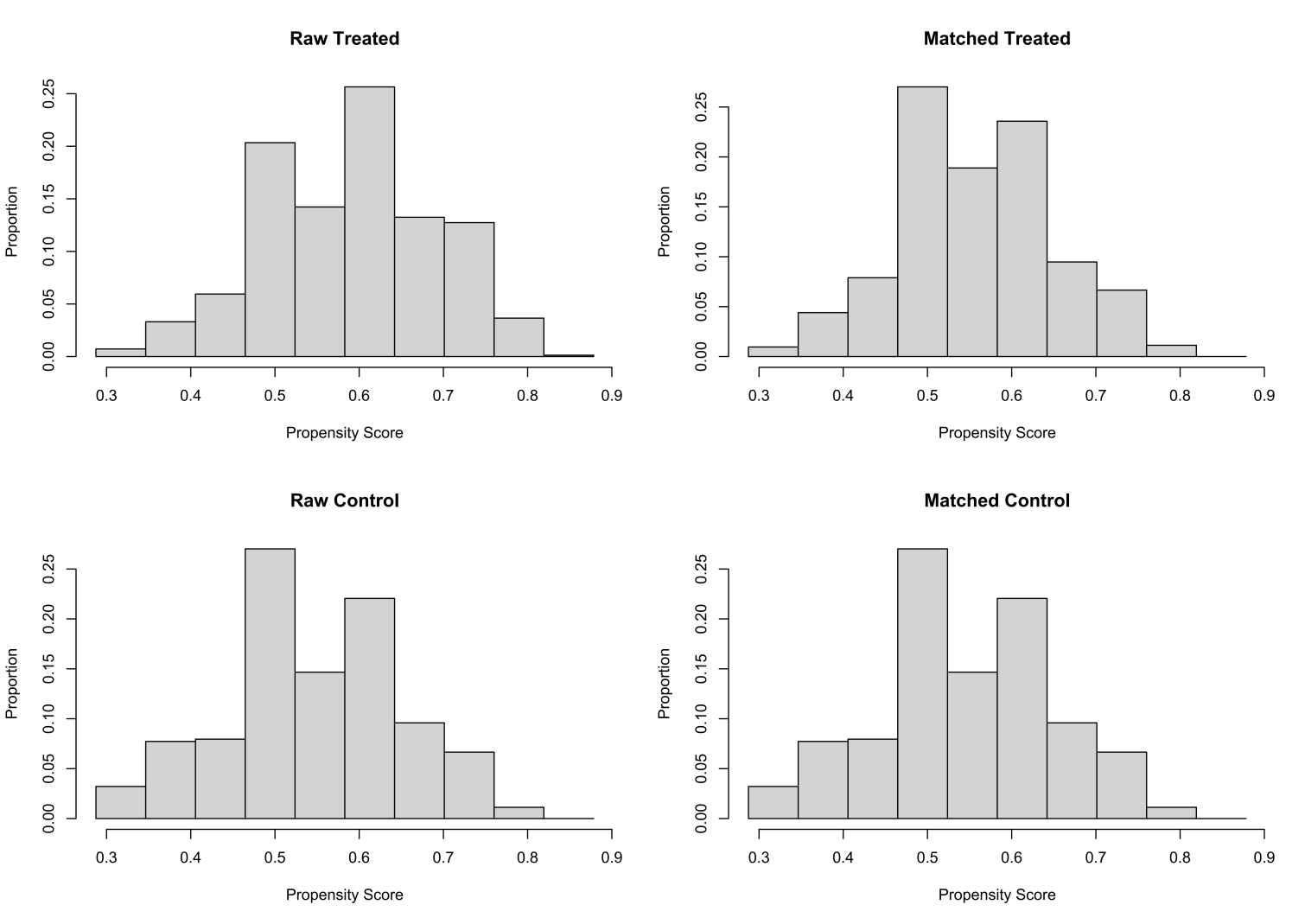
**

**Figure S2** Propensity score distribution before and after matching

**Table S1** Comparison of demographic and lifestyle characteristics between the 2024 and 2025 survey waves

|  | Wave 2024 | Wave 2025 | *P* |
| --- | --- | --- | --- |
| Age; mean±SD | 38.79±11.13 | 25.18±5.96 | <0.001 |
| Sex; n(%) |  |  | <0.001 |
| Male | 993(56.68) | 1016(42.76) |  |
| Female | 759(43.32) | 1360(57.24) |  |
| Ethnicity; n(%) |  |  | <0.001 |
| Han | 1713(97.77) | 2114(88.97) |  |
| Others | 39(2.23) | 262(11.03) |  |
| Residence; n(%) |  |  | <0.001 |
| Urban | 1264(72.15) | 1321(55.60) |  |
| Rural | 488(27.85) | 1055(44.40) |  |
| Educational level; n(%) |  |  | <0.001 |
| Junior high or below | 138(7.88) | 2(0.08) |  |
| Senior high | 365(20.83) | 50(2.10) |  |
| College or above | 1249(71.29) | 2324(97.81) |  |
| Overweight/obesity; n(%) |  |  | 0.297 |
| No | 1341(76.54) | 1784(75.08) |  |
| Yes | 411(23.46) | 592(24.92) |  |
| Smoking; n(%) |  |  | <0.001 |
| No | 1224(69.86) | 2050(86.28) |  |
| Yes | 528(30.14) | 326(13.72) |  |
| Drinking; n(%) |  |  | <0.001 |
| No | 1030(58.79) | 806(33.92) |  |
| Yes | 722(41.21) | 1570(66.08) |  |
| Regular physical exercise; n(%) |  |  | <0.001 |
| No | 978(55.82) | 1984(83.50) |  |
| Yes | 774(44.18) | 392(16.50) |  |

**Table S2** Association between dietary diversity and PHQ-9 scores/depression

|  | Before propensity 1:1 matching | | | | After propensity 1:1 matching | | | |
| --- | --- | --- | --- | --- | --- | --- | --- | --- |
|  | PHQ-9 scores | | Depression | | PHQ-9 scores | | Depression | |
|  | β(95% CI) | aβ(95% CI)^#^ | OR(95% CI) | aOR(95% CI)^#^ | β(95% CI) | aβ(95% CI)^#^ | OR(95% CI) | aOR(95% CI)^#^ |
| Total DDS, per 1-score increase | -0.66(-0.74,-0.57)^***^ | -0.62(-0.71,-0.54)^***^ | 0.74(0.71,0.78)^***^ | 0.75(0.71,0.79)^***^ | -0.64(-0.72,-0.55)^***^ | -0.63(-0.73,-0.54)^***^ | 0.73(0.68,0.79)^***^ | 0.77(0.70,0.84)^***^ |
| Animal-based DDS, per 1-score increase | -1.09(-1.23,-0.94)^***^ | -1.01(-1.16,-0.86)^***^ | 0.58(0.54,0.63)^***^ | 0.61(0.56,0.66)^***^ | -1.06(-1.21,-0.90)^***^ | -1.03(-1.19,-0.87)^***^ | 0.60(0.52,0.68)^***^ | 0.63(0.54,0.73)^***^ |
| Plant-based DDS, per 1-score increase | -0.85(-1.00,-0.71)^***^ | -0.79(-0.93,-0.65)^***^ | 0.72(0.66,0.77)^***^ | 0.72(0.67,0.79)^***^ | -0.82(-0.96,-0.67)^***^ | -0.80(-0.95,-0.65)^***^ | 0.68(0.60,0.78)^***^ | 0.73(0.64,0.84)^***^ |
| AIDDI, per 1-score increase | -0.70(-0.81,-0.58)^***^ | -0.66(-0.78,-0.54)^***^ | 0.76(0.71,0.81)^***^ | 0.76(0.71,0.81)^***^ | -0.65(-0.78,-0.53)^***^ | -0.66(-0.79,-0.54)^***^ | 0.72(0.65,0.81)^***^ | 0.77(0.69,0.87)^***^ |
| PEDDI, per 1-score increase | -0.69(-0.80,-0.59)^***^ | -0.64(-0.74,-0.53)^***^ | 0.71(0.68,0.75)^***^ | 0.74(0.69,0.78)^***^ | -0.67(-0.78,-0.57)^***^ | -0.65(-0.76,-0.54)^***^ | 0.73(0.66,0.80)^***^ | 0.76(0.68,0.84)^***^ |

PHQ-9:Patient Health Questionnaire-9; DDS: Dietary Diversity Score; AIDDI: Anti-inflammatory Dietary Diversity Index; PEDDI: Protein-enriched Dietary Diversity Index; #: Adjusted for age, sex, ethnicity, residence, educational level, overweight/obesity, smoking, drinking, regular physical exercise and wave; ^***^: *P*<0.001.

**Table S3** Sensitivity analysis 1: Association between dietary diversity and PSQI scores/poor SQ stratified by survey wave

|  | PSQI scores, β(95% CI)^#^ | | Poor SQ, OR(95% CI)^#^ | |
| --- | --- | --- | --- | --- |
|  | Wave 2024 | Wave 2025 | Wave 2024 | Wave 2025 |
| Total DDS, per 1-score increase | -0.92(-1.11,-0.74)^***^ | -0.19(-0.25,-0.13)^***^ | 0.81(0.76,0.86)^***^ | 0.88(0.83,0.92)^***^ |
| Animal-based DDS, per 1-score increase | -1.33(-1.64,-1.03)^***^ | -0.28(-0.39,-0.17)^***^ | 0.78(0.71,0.86)^***^ | 0.82(0.74,0.90)^***^ |
| Plant-based DDS, per 1-score increase | -1.28(-1.61,-0.95)^***^ | -0.27(-0.37,-0.17)^***^ | 0.70(0.63,0.77)^***^ | 0.84(0.77,0.91)^***^ |
| AIDDI, per 1-score increase | -1.06(-1.33,-0.78)^***^ | -0.23(-0.31,-0.15)^***^ | 0.73(0.67,0.80)^***^ | 0.86(0.81,0.92)^***^ |
| PEDDI, per 1-score increase | -0.84(-1.06,-0.63)^***^ | -0.18(-0.26,-0.11)^***^ | 0.83(0.78,0.89)^***^ | 0.87(0.82,0.93)^***^ |

PSQI: Pittsburgh sleep quality index; SQ: Sleep quality; DDS: Dietary Diversity Score; AIDDI: Anti-inflammatory Dietary Diversity Index; PEDDI: Protein-enriched Dietary Diversity Index; #: Adjusted for age, sex, ethnicity, residence, educational level, overweight/obesity, smoking, drinking, and regular physical exercise; ^***^: *P*<0.001.

**Table S4** Sensitivity analysis 2: Association between dietary diversity and MEQ-5 scores/chronotype stratified by survey wave

|  | MEQ-5 scores,  aβ(95% CI)^#^ | | Intermediate type vs. Morning type, aOR(95% CI)^#^ | | Evening type vs. Morning type,  aOR(95% CI)^#^ | |
| --- | --- | --- | --- | --- | --- | --- |
|  | Wave 2024 | Wave 2025 | Wave 2024 | Wave 2025 | Wave 2024 | Wave 2025 |
| Total DDS, per 1-score increase | 0.27(0.19,0.36)^***^ | 0.26(0.18,0.34)^***^ | 0.83(0.78,0.89)^***^ | 0.80(0.74,0.87)^***^ | 0.75(0.67,0.84)^***^ | 0.73(0.67,0.80)^***^ |
| Animal-based DDS, per 1-score increase | 0.30(0.17,0.44)^***^ | 0.30(0.16,0.44)^***^ | 0.77(0.69,0.85)^***^ | 0.68(0.59,0.79)^***^ | 0.76(0.64,0.91)^**^ | 0.64(0.54,0.75)^***^ |
| Plant-based DDS, per 1-score increase | 0.49(0.34,0.63)^***^ | 0.43(0.31,0.56)^***^ | 0.78(0.70,0.87)^***^ | 0.76(0.67,0.86)^***^ | 0.57(0.47,0.70)^***^ | 0.63(0.55,0.73)^***^ |
| AIDDI, per 1-score increase | 0.39(0.27,0.51)^***^ | 0.39(0.29,0.50)^***^ | 0.82(0.75,0.90)^***^ | 0.77(0.70,0.85)^***^ | 0.63(0.54,0.74)^***^ | 0.66(0.59,0.75)^***^ |
| PEDDI, per 1-score increase | 0.22(0.12,0.31)^***^ | 0.23(0.14,0.33)^***^ | 0.82(0.77,0.89)^***^ | 0.79(0.71,0.87)^***^ | 0.82(0.72,0.93)^**^ | 0.74(0.66,0.82)^***^ |

PSQI: Pittsburgh sleep quality index; SQ: Sleep quality; DDS: Dietary Diversity Score; Ab DDS: Animal-based Dietary Diversity Score; Pb DDS: Plant-based Dietary Diversity Score; AIDDI: Anti-inflammatory Dietary Diversity Index; PEDDI: Protein-enriched Dietary Diversity Index; #: Adjusted for age, sex, ethnicity, residence, educational level, overweight/obesity, smoking, drinking, and regular physical exercise; ^**^: *P*<0.01; ^***^: *P*<0.001.

**Table S5** Sensitivity analysis 3: Association between dietary diversity and PHQ-9 scores/depression stratified by survey wave

|  | PHQ-9 scores, aβ(95% CI)^#^ | | Depression, aOR(95% CI)^#^ | |
| --- | --- | --- | --- | --- |
|  | Wave 2024 | Wave 2025 | Wave 2024 | Wave 2025 |
| Total DDS, per 1-score increase | -0.90(-1.02,-0.77)^***^ | -0.39(-0.51,-0.27)^***^ | 0.64(0.59,0.69)^***^ | 0.86(0.81,0.92)^***^ |
| Animal-based DDS, per 1-score increase | -1.43(-1.63,-1.22)^***^ | -0.58(-0.80,-0.36)^***^ | 0.48(0.42,0.54)^***^ | 0.79(0.70,0.89)^***^ |
| Plant-based DDS, per 1-score increase | -1.09(-1.32,-0.86)^***^ | -0.56(-0.75,-0.37)^***^ | 0.62(0.54,0.70)^***^ | 0.82(0.74,0.92)^***^ |
| AIDDI, per 1-score increase | -0.89(-1.08,-0.70)^***^ | -0.47(-0.63,-0.31)^***^ | 0.67(0.60,0.75)^***^ | 0.85(0.77,0.94)^***^ |
| PEDDI, per 1-score increase | -0.91(-1.06,-0.76)^***^ | -0.39(-0.54,-0.25)^***^ | 0.63(0.58,0.69)^***^ | 0.85(0.79,0.93)^***^ |

PSQI: Pittsburgh sleep quality index; SQ: Sleep quality; DDS: Dietary Diversity Score; AIDDI: Anti-inflammatory Dietary Diversity Index; PEDDI: Protein-enriched Dietary Diversity Index; #: Adjusted for age, sex, ethnicity, residence, educational level, overweight/obesity, smoking, drinking, and regular physical exercise; ^***^: *P*<0.001.

**Table S6** Sensitivity analysis 4: Association between dietary diversity and poor SQ defined as a PSQI score >5

|  | Before pooling waves, aOR(95% CI)^#^ | | After pooling waves, aOR(95% CI)^#^ | |
| --- | --- | --- | --- | --- |
|  | Wave 2024 | Wave 2025 | Before propensity 1:1 matching | After propensity 1:1 matching |
| Total DDS, per 1-score increase | 0.85(0.80,0.91)^***^ | 0.76(0.68,0.85)^***^ | 0.82(0.78,0.87)^***^ | 0.77(0.67,0.87)^***^ |
| Animal-based DDS, per 1-score increase | 0.86(0.78,0.96)^*^ | 0.68(0.55,0.83)^***^ | 0.82(0.75,0.90)^***^ | 0.71(0.56,0.88)^**^ |
| Plant-based DDS, per 1-score increase | 0.72(0.64,0.81)^***^ | 0.68(0.57,0.80)^***^ | 0.70(0.63,0.77)^***^ | 0.67(0.54,0.82)^***^ |
| AIDDI, per 1-score increase | 0.74(0.68,0.82)^***^ | 0.74(0.64,0.85)^***^ | 0.73(0.68,0.79)^***^ | 0.72(0.61,0.86)^***^ |
| PEDDI, per 1-score increase | 0.88(0.82,0.95)^***^ | 0.75(0.65,0.85)^***^ | 0.85(0.79,0.90)^***^ | 0.80(0.69,0.93)^**^ |

PSQI: Pittsburgh sleep quality index; SQ: Sleep quality; DDS: Dietary Diversity Score; AIDDI: Anti-inflammatory Dietary Diversity Index; PEDDI: Protein-enriched Dietary Diversity Index; #: Adjusted for age, sex, ethnicity, residence, educational level, overweight/obesity, smoking, drinking, regular physical exercise; ^*^: *P*<0.05; ^**^: *P*<0.01; ^***^: *P*<0.001.
